# Molecular features and vulnerabilities of recurrent chordomas

**DOI:** 10.1186/s13046-021-02037-y

**Published:** 2021-07-30

**Authors:** Carolin Seeling, André Lechel, Michael Svinarenko, Peter Möller, Thomas F. E. Barth, Kevin Mellert

**Affiliations:** 1 Institute of Pathology, University Hospital Ulm, Albert-Einstein-Allee 11, 89081 Ulm, Germany; 2 Department of Internal Medicine I, University Hospital Ulm, 89081 Ulm, Germany

**Keywords:** Chordoma, Cell lines, Progression model, HOX, PBX, Apoptosis

## Abstract

**Background:**

Tumor recurrence is one of the major challenges in clinical management of chordoma. Despite R0-resection, approximately 50% of chordomas recur within ten years after initial surgery. The underlying molecular processes are poorly understood resulting in the lack of associated therapeutic options. This is not least due to the absence of appropriate cell culture models of this orphan disease.

**Methods:**

The intra-personal progression model cell lines U-CH11 and U-CH11R were compared using array comparative genomic hybridization, expression arrays, RNA-seq, and immunocytochemistry. Cell line origin was confirmed by short tandem repeat analysis. Inter-personal cell culture models (*n* = 6) were examined to validate whether the new model is representative. Cell viability after HOX/PBX complex inhibition with small peptides was determined by MTS assays.

**Results:**

Using whole genome microarray analyses, striking differences in gene expression between primary and recurrent chordomas were identified. These expression differences were confirmed in the world’s first intra-personal model of chordoma relapse consisting of cell lines established from a primary (U-CH11) and the corresponding recurrent tumor (U-CH11R). Array comparative genomic hybridization and RNA-sequencing analyses revealed profound genetic similarities between both cell lines pointing to transcriptomic reprogramming as a key mechanism of chordoma progression. Network analysis of the recurrence specific genes highlighted HOX/PBX signaling as a common dysregulated event. Hence, HOX/PBX complexes were used as so far unknown therapeutic targets in recurrent chordomas. Treating chordoma cell lines with the complex formation inhibiting peptide HXR9 induced cFOS mediated apoptosis in all chordoma cell lines tested. This effect was significantly stronger in cell lines established from chordoma relapses.

**Conclusion:**

Clearly differing gene expression patterns and vulnerabilities to HOX/PBX complex inhibition in highly therapy resistant chordoma relapses were identified using the first intra-personal loco-regional and further inter-personal chordoma progression models. For the first time, HOX/PBX interference was used to induce cell death in chordoma and might serve as the basic concept of an upcoming targeted therapy for chordomas of all progression stages.

**Supplementary Information:**

The online version contains supplementary material available at 10.1186/s13046-021-02037-y.

## Background

Chordomas are slow growing but locally invasive tumors of the axial skeleton [1]. Microscopically, strongly vacuolated cells, so called physaliferous cells, are diagnostically indicative for this lesion [2].

Chordoma cells are thought to originate from cellular remnants of the notochord, as the most consistent feature is the sustained expression of the developmental transcription factor brachyury. Beyond being a sensitive chordoma marker, high levels of nuclear brachyury, encoded by the *TBXT* gene, appear to significantly contribute to tumorigenesis. Thus, shRNA- and sgRNA-mediated *TBXT* repression in chordoma cell lines have been shown to suppress cell growth and induce apoptosis [3, 4]. Moreover, germline duplication of *TBXT* is associated with chordoma risk in rare familiar cases, whereas the nonsynonymous SNP rs2305089 can be also found in sporadic chordomas and has prognostic significance in overall survival [5, 6]. Further genomic aberrations of chordomas include mutations in SWI/SNF chromatin modelling genes (*PBRM1*, *SMARCB1*), PI3K signaling genes (*PIK3CA*, *PTEN*) and the *LYST* gene [7]. Somatic copy number alterations involve gains of chromosome 7p and homozygous deletions of chromosome 9p21 region, harboring the *EGFR* and *CDKN2A* gene loci, respectively [8]. Recently, deletions of 9p21.3, 9p21.11 and 22q deletion as well as SWI/SNF alterations have been associated to shorter recurrence free survival in skull base chordomas [9]. However, generally, only few genes are recurrently mutated and the full mutational landscape in chordoma is not known, yet. Due to their slow growth chordomas are considered chemo- and radio-resistant [10]. Based on the identification of potential molecular drivers in chordoma and the implementation of small molecule library screenings, selected small molecules, such as Palbociclib (NCT03110744) or Afatinib (NCT03083678), targeting p16 and EGFR, respectively, are currently evaluated in phase 2 clinical trials. However, so far there are no phase 3 clinical trials or approved anticancer drugs. Therefore, curative *en-bloc* resection is the recommended treatment for primary tumors whenever feasible. For incompletely resected chordomas an adjuvant high-dose radiotherapy should be considered [11]. Though, loco-regional tumor recurrence following initial *en-bloc* resection with or without subsequent radiation is high. Loco-regional recurrence rates of > 50% were published [12]. Notably, the risk of recurrence varies depending on the localization of the tumor with the highest rates observed for sacral chordomas [13]. Although tumor free surgical resection margins are regarded as the most important predictor of local chordoma recurrence, the risk of local recurrence remains high even when the resection is initially considered R0 [12]. Completely resected primary, sacral chordomas have a 5- and 10-year local relapse rate of approximately 25 and 50%, respectively [14].

Despite the high propensity to relapse, little is known about the underlying molecular processes accounting for chordoma progression. This is probably due to the rareness of the disease (incidence< 0.1:100000) and the deficiency of appropriate cell culture models [15].

Here, we investigated the molecular differences in primary and recurrent chordomas using the cell lines U-CH11 and U-CH11R, which were established from the primary and the recurrent sacral chordoma of the same patient. We identified targetable genetic features of U-CH11R and inhibited the dimerization of PBX and the HOX transcription factors to induce apoptosis. This approach was extended to further chordoma cell lines.

## Methods

### Establishment of chordoma cell lines and cells in culture

The establishment and maintenance of chordoma cell lines has been previously described [13]. The chordoma cell lines U-CH1, U-CH2, U-CH11, U-CH11R, U-CH17P, U-CH19, and MUG-Chor1 were included and cultured as previously described [8, 13, 16–19]. Human foreskin fibroblasts (HFF) from three different donors were used as control cells. Cell lines were quality controlled by short tandem repeat (STR) analysis using the GenomeLab STR Primer Set Kit (Beckman Counter, Krefeld, Germany) and were tested regularly for mycoplasma contamination via PCR as described earlier (latest report in Additional file 1 (Additional Figure 1)) [18]. Patients gave their informed written consent and the study was in line with the Declaration of Helsinki and approved by the local ethics committee (vote 369/17).

### Cell proliferation and cell viability assay

Population doubling time (PDT) and cell viability were assessed by MTS assays (Abcam, Cambridge, UK) according to the manufacturer’s recommendations. To determine the PDT of U-CH11 and U-CH11R, cells were seeded in 96-well culture plates at 7500 cells/cm^2^ and 15,000 cells/cm^2^ in biological and technical triplicates. Cell viability was examined at different time points up to 336 h. PDT was calculated by GraphPad Prism v.5 (GraphPad Software, Inc., San Diego, CA, USA) using a best fit exponential growth equation.

For cell viability assays in response to HXR9 and CXR9, cells were seeded in 96-well culture plates and allowed to adhere overnight, following the incubation of the peptides for 24 h. The amino acid sequences of HXR9 and CXR9 have been previously published by Morgan et al. [20, 21]. The peptides were custom synthesized by Biosynthesis Inc. (Lewisville, TX, USA) using conventional column-based chemistry and purified to > 90%. IC_50_ values were determined using GraphPad Prism v.5. All cell lines were tested in biological and technical triplicates.

### Colony formation in soft agar

The colony formation assay was performed as previously described [18]. Plated monolayer U-CH1, U-CH19, U-CH11, and U-CH11R cells were treated with 50 μM HXR9 or CXR9 for 8 h prior to replating 10,000 cells/well in soft agar base in six-well dishes. Cells were maintained at 37 °C for 32 days and growth medium was replaced twice a week. Number of colonies was counted using the ImageJ software (NIH). For each cell line three independent experiments were performed.

### Array comparative genomic hybridization

Array comparative genomic hybridization (aCGH) was performed with the SurePrint G3 Human CGH Microarray 8 × 60 (Agilent Technologies) as described previously [13]. Array-CGH data were analyzed using the Genomics Workbench software (Agilent Technologies) applying the ADM-2 aberration algorithm (threshold 6.0; cutoff ±0.5).

### RNA-sequencing analysis

RNA-sequencing data of the primary chordoma tissue as well as U-CH11 and U-CH11R cell lines was obtained from the Chordoma Foundation (https://www.chordomafoundation.org). The GATK workflow for calling single nucleotide polymorphisms (SNPs) and indels in RNA-sequencing experiments was used (https://gatkforums.broadinstitute.org/gatk/discussion/3891/calling-variants-in-rnaseq). Raw reads were processed with Trimmomatic (v0.39) to remove adapter sequences and aligned with the STAR (v2.7) 2-pass method against the human genome build37 (hg19) [22–24]. Variants were called and filtered by the GATK-HaplotypeCaller (v4.1.4.1).

### Variant filtering and SNP selection

The functional annotation of the variants was performed using ANNOVAR [25]. Only exonic variants with a read depth ≥ 20 were included in further analyses and selected on the basis of allele frequency (AF < 1%) obtained from the genome Aggregation Database v2 (gnomAD) and of genomic function (nonsynonymous SNV, frameshift deletion, frameshift insertion, startloss, stopgain).

### Microarray gene expression profiling

Microarray gene expression profiling was performed as described earlier [13]. Data was processed and analyzed using the GeneSpring 14.9 software (*p* < 0.05, FC > 2). Each cell line was tested at least in duplicates. Basal expression levels of selected genes are given (Additional file 1 (Additional Table 8)).

### Real-time polymerase chain reaction (qRT-PCR)

Gene expression analysis of *cFOS* and verification of selected microarray results was performed using qRT-PCR. RNA extraction was performed with the RNeasy Mini-Kit (Qiagen, Hilden, Germany). Total RNA was reverse transcribed to cDNA using the SuperScript IV Reverse Transcriptase Kit (ThermoFisher Scientific, Waltham, MA, USA). Gene expression was quantified by qRT-PCR utilizing the QuantiTect SYBR Green Master Mix (Qiagen) and uniquely designed gene expression primers (*CFOS*, *GABRA2*, *GYLAT*, *SOST*, *HOXA3*, *HOXB7,* and *HOXB13*) including *GAPDH* and *ACTB* reference genes. Analyses were performed in technical triplicates and in at least biological duplicates.

### miRNA expression analysis

miRNA was extracted using the miRNeasy Mini Kit (Qiagen) according to the manufacturer’s handbook. For mature miRNA expression analysis, cDNA synthesis of 1 μg total RNA was carried out using the miScript Reverse Transcription Kit (Qiagen) with HighFlex buffer. qRT-PCR was performed using the QuantiNova SYBR Green PCR Kit and the Light-cycler Rotor Gene Q (Qiagen). The miScript Primer Assay for the mature Hs-miR-196a_2, Hs-miR-196b_2 and mirScript Universal Primer (all obtained from Qiagen) were used according to manufacturer’s protocol. Relative miRNA levels were normalized to RNU6_2 snRNA. All measurements were performed in technical and biological triplicates. Relative fold changes were calculated using the comparative cycle threshold (Ct) eq. (2^-△△Ct^).

### Luciferase assay

Luciferase firefly pIS0-vectors containing HOXA7–3’UTR, HOXB8–3’UTR, and mir196a-as were a gift from David Bartel (purchased from Addgene [26];). Adherent HEK293T and U-CH1 were grown in 10% FCS supplemented IMDM to 90% confluency in 24-well plates. Cells were cotransfected with 0.4 μg of the firefly luciferase vector, 0.08 μg of the control vector containing Renilla luciferase (pRL-SV40P, Addgene, Quelle: Serum-induced expression of the cdc25A gene relief of E2F-mediated repression) and 2 pmol of synthetic miRNA-196a-5p mimic (abm, Richmond, BC, Canada) or a scrambled control microRNA in a final volume of 0.25 ml using Lipofectamine 2000 (ThermoFisher Scientific). Firefly and Renilla luciferase activities were measured consecutively 24 h after transfection using the Dual-Glo Luciferase Assay System and a luminometer (FB12 Luminometer, Titertek-Berthold, Pforzheim, Germany).

### MicroRNA mimic and hairpin inhibitor transfection

Chordoma cell lines were grown in 10% FCS supplemented IMDM to 90% confluency in 6-well plates and allowed to adhere overnight. Cells were then transfected with 100 nM miR-196a-5p mimic (Dharmacon) or scrambled sequence (abm, Richmond, BC, Canada) by using Lipofectamine 2000 (ThermoFisher Scientific) according to the manufacturer’s protocol and harvested after 6 h for Western blot and qRT-PCR analyses.

### Immunohistochemistry and immunocytochemistry

Immunostaining of formalin-fixed and paraffin-embedded cell blocks and tissue sections (4 μm) were carried out by the avidin-biotin-complex method applying the K005 AP/RED Detection System (Dako, Glostrup, Denmark). The antibodies used are given in Additional file 1 (Additional Table 1). The ratio of positive cells was annotated as follows: “no immunoreactivity detected” (−), “immunoreactivity ≤30%” (+), “immunoreactivity >30% and <70%” (++), “immunoreactivity ≥70%” (+++).

### Immunoblotting

Cell pellets were resuspended in RIPA lysis buffer containing protease and phosphatase inhibitor (ThermoFisher Scientific). Lysates were incubated in liquid nitrogen for 3 min, thawed and then centrifuged at 14000 rpm and 4 °C to remove debris. Protein quantification of the supernatants was performed using the Pierce BCA Protein Assay Kit (ThermoFisher Scientific). Prior Western blot analyses samples were equalized for protein amount, reduced with DTT and denatured at 90 °C for 5 min. Protein samples were resolved using 4–12% Bis-Tris gels (ThermoFisher Scientific) and then transferred to nitrocellulose membranes using a wet transfer device. Membranes were probed with primary antibodies at 4 °C overnight.

To assess apoptosis in chordoma cell lines following a 24 h treatment with HXR9 and CXR9, cleaved PARP (Asp214), cleaved Caspase-7 (Asp198; both 1:1000; Cell Signaling; Cambridge, UK) and a cocktail of primary antibodies to detect apoptosis biomarkers pro/p17-Caspase-3, cleaved Caspase-3, along with ß-Actin loading control (abcam, Cambridge, UK) were used.

Protein lysates of miRNA mimic transfected cells were analyzed using an antibody against HOXA7 (1:1000; clone 2F2; Abnova, Taipeh, Taiwan) and ß-Actin (1:2000; BA3R clone; ThermoFisher Scientific).

Membranes were incubated with secondary antibodies (Goat anti-Mouse IgG (H + L) 1:10000, ThermoFisher Scientific; Goat anti-Rabbit-IgG (whole molecule) 1:2000, Sigma-Aldrich, St. Louis, USA) and detected with the WesternSure Chemiluminescent Substrate (LI-CORE Biosciences, Lincoln, NE, USA). Immunoblots were quantified by densitometry using the ImageJ software (NIH).

### Caspase-3/7 activity assay

Caspase 3/7 activation in cells was assessed using the EarlyTox Caspase3/7 NucView 488 kit (Molecular devices, UK) following the manufacture’s instructions. Chordoma cells were plated at approx. 2500 cells/chamber in an 8-well Nunc Lab-Tek II CC2 chamber slide system (ThermoFisher Scienfic) and allowed to adhere overnight. HXR9 and CXR9 peptides (30 μM) were added to the cells and incubated for 24 h. The Caspase-3/7 NucView was then added directly to the cells at a final concentration of 5 μM and incubated at room temperature for 30 min, protected from light. Imaging was performed on the PAULA Cell imager (Leica Microsystems, Wetzlar, Germany).

### Rank-rank hypergeometric overlap analysis

The overlap trend between two complete, continuous gene-expression signatures was identified and visualized using the rank-rank hypergeometric overlap (RRHO) algorithm (https://systems.crump.ucla.edu/rankrank/rankranksimple.php). Genes were ranked by their log_10_-transformed t-test *p*-values and effect size direction; the Benjamini and Yekutieli method was applied as a multiple hypothesis correction factor. A rank-rank overlap heatmap was generated to visualize the range of gene expression overlap between two signatures. The strength of enrichment is indicated as -log_10_ transformed hypergeometric *p*-value [27].

### Functional enrichment analyses

Gene set enrichment analyses (GSEA) were performed by computing overlaps between identified class-specific gene signatures and gene sets derived from the Molecular Signature Database (MSigDB) v7.1 [28]. For statistical computing, the RStudio v1.2.5 software and the R ClusterProfiler package were used applying the hypergeometric distribution method. The following thresholds were applied to determine statistical significance: Benjamin&Hochberg adjusted p-value < 0.05, q-value < 0.05 [29]. The enrichplot v1.6.1 package was implemented to visualize functional enrichment results obtained from the GSEA. Protein-protein interactions were predicted by STRING v11.0 database analysis applying a minimum required interaction score of 0.4 [30].

### miRNA target gene predictions

Micro-RNA target genes of the miR-196a-5p were predicted using TargetScan v7.2 (http://www.targetscan.org/vert_72/), miRDB v6.0 (www.mirdb.org) and mirDIP v4.1 (http://ophid.utoronto.ca/mirDIP/).

### Statistical analysis

For statistical analyses Student’s t-tests were performed. A *p*-value ≤0.05 was considered significant.

## Results

### Clinical history of the tumor progression model

The cell lines U-CH11 and U-CH11R were established from the primary and the corresponding recurrent chordoma of a 71-year-old male Caucasian patient. The initial lesion was located at the sacrum and was 8 × 5.5 × 3 cm in size. Following a surgical resection, considered R0, the chordoma locally relapsed within 40 months after the first diagnosis and measured 6.5 × 5.5 × 4 cm at the time of re-excision. Clonal cell selection and genetic alterations induced upon radio- or chemotherapy can be excluded, as this was no part of the patient’s treatment regimen.

### Characterization of the recurrent lesion and the derived cell line U-CH11R

**Fig. 1:**
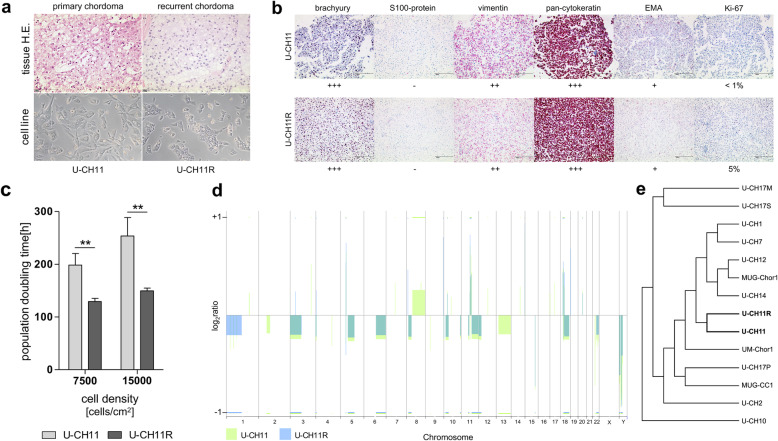
U-CH11 and U-CH11R cells are clonally related. **a** Hematoxylin/eosin staining of the primary and the recurrent chordoma tissues and in vitro light microscopy of U-CH11 and U-CH11R. The physaliferous cell morphology is conserved. **b** Immunocytochemical staining of the chordoma markers brachyury, S100-protein, vimentin, pan-cytokeratin, epithelial membrane antigen (EMA), and Ki-67. **c** Mean in vitro population doubling times (±SD; *n* = 3) of U-CH11 and U-CH11R at different cell densities (**p < 0.01) **d** Overlay of the array comparative genomic hybridization ratio plots of U-CH11 (green) and U-CH11R (blue) showing a high concordance of alterations. **e** Hierarchical cluster analysis of U-CH11 and U-CH11R in comparison to 12 additional chordoma cell lines. Clustering was performed in a Euclidean distance measure and single linkage rule

Both, primary and recurrent tumors show nuclear brachyury expression and were histologically classified as classical, physaliferous chordomas (not otherwise specified, NOS) according to the WHO criteria. The cell line U-CH11 was characterized previously [8]. The cell line U-CH11R was authenticated by comparing its STR profile with the profile of U-CH11 (Additional file 1 (Additional Table 2)). In concordance with the originating tumor tissue, cells of U-CH11R feature the typical appearance of chordomas including a strongly vesiculated cytoplasm and nuclear brachyury expression. (Fig. 1a and b).

The immunocytochemical chordoma panel of the cell line largely represents the panel of the fixed tumor tissue (Additional file 1 (Additional Figure 2)). U-CH11 and U-CH11R exhibit very similar immunocytological profiles with both being positive for pan-cytokeratin and vimentin, weakly positive for the epithelial membrane antigen (EMA), and negative for S100-protein. Differences were seen regarding the Ki-67 labeling index, which was estimated 1% in U-CH11 and 5% in U-CH11R (Fig. 1b).

Consistent with the higher proliferation index, the population doubling time (PDT) of U-CH11R was significantly shorter (student’s t-test; **p < 0.01) (Fig. 1c).

### U-CH11 and U-CH11R cells are genetically similar

In order to assess the clonal relationship between U-CH11 and U-CH11R cells, the genomic aberration profiles of the two cell lines were determined by aCGH. A high concordance of chromosomal alterations between both cell lines was observed, including mainly losses on chromosomes 3p, 5q, 6q, 8p, 10p, 11q, 12p, 18q, 22q, and Y. Additionally, gains of chromosomal material were detected on 11q, 18q, and 20q. Only few chromosomes were altered differently, such as an extra gain of 8q and loss of 13q in U-CH11 and a loss of 1p in U-CH11R (Fig. 1d).

In an unsupervised hierarchical clustering based on the aCGH data of 12 additional chordoma cell lines, U-CH11 and U-CH11R formed a distinct subgroup, substantiating the close relation between these two cell lines (Fig. 1e).

### Mutations in the primary chordoma tissue are conserved in U-CH11 and U-CH11R

**Fig. 2:**
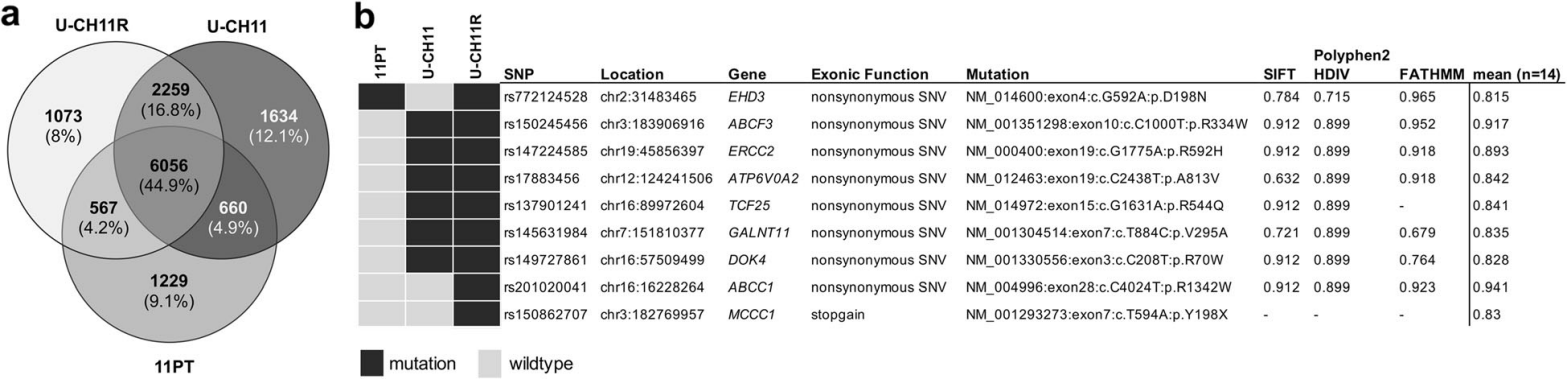
RNA-Sequencing. **a** Venn diagram illustrating the mutational concordance of exonic variants in the primary chordoma tissue (11PT), the derived primary cell line U-CH11 and the recurrent chordoma cell line U-CH11R. **b** Deleterious mutations observed in the samples (colour coded, refer to legend). SIFT, Polyphen2-HDIV and FATHMM represent three examples of all implemented SNP analysis tools (*n* = 14)

Genetic alterations in the U-CH11 and U-CH11R cell lines in relation to the primary chordoma tissue were investigated by RNA-sequencing. Approximately 80% (6725/8512) of the exonic SNPs detected in the primary chordoma tissue were conserved in the derived primary chordoma cell line U-CH11 (Fig. 2a).

A large proportion of the in common mutations was also detected in the recurrent chordoma derived cell line U-CH11R (90%; 6056/6725). Furthermore, a number of variants (30%; 567/1796) in the tissue that were not detected in U-CH11 were found in U-CH11R supporting the hypothesis that a cell clone within the primary chordoma gave rise to the recurrent tumor. All samples harbored the brachyury Gly177Asp SNP (rs2305089) which is strongly associated with chordoma in the European population [31].

Exonic alterations were further filtered by allele frequency and genomic consequence. The mean rankscore of various SNP analysis tools was calculated to predict the deleteriousness of any amino acid change. The score ranges from 0 to 1 and variants with higher risks are predicted to be more likely pathogenic. A cut-off of 0.8 was used to filter the most likely deleterious substitutions. Figure 2b gives an overview of the discovered mutations (Full list: Additional file 2). In the primary chordoma tissue, a pathogenic mutation in *EHD3* (rs772124528) was detected, which was also found in U-CH11R. Two additional mutations in *ABCC1* (rs201020041) and *MCCC1 (*rs150862707) were exclusively found in U-CH11R. Moreover, a number of pathogenic mutations, such as in *ABCF3*, were detected in both, U-CH11 and U-CH11R, but not in the tumor tissue.

### Microarray gene expression analysis reveals significant differences between U-CH11 and U-CH11R

**Fig. 3:**
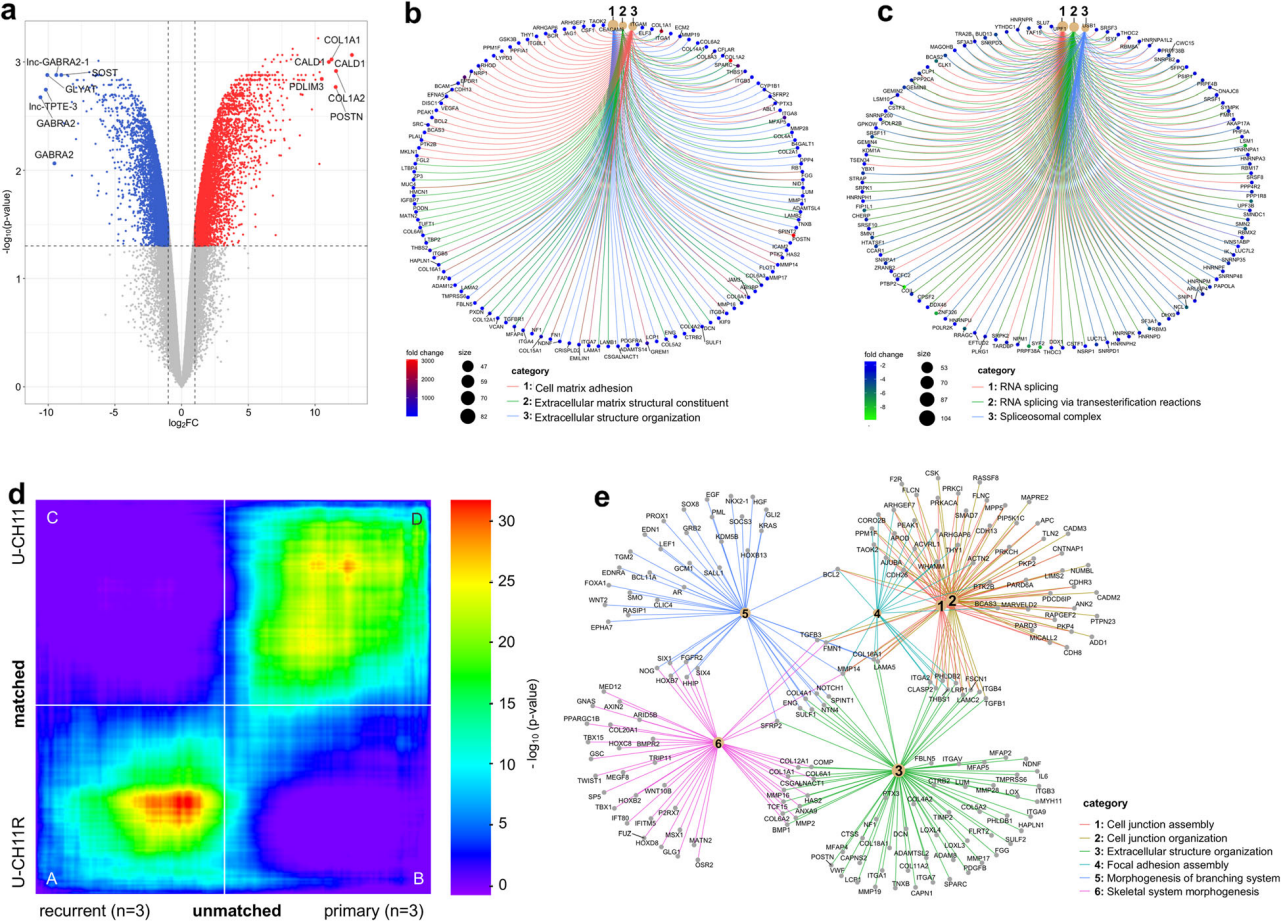
U-CH11 and U-CH11R present distinct transcriptomic profiles representative for sacral primary and recurrent chordoma lines. **a** Volcano plot showing statistical significance (−log_10_*p*-value) versus fold change (log_2_FC) of microarray gene expression data from U-CH11R (n = 3) versus U-CH11 (*n* = 2). The top five diminished (blue) and enhanced (red) genes in U-CH11R are annotated. Cnetplots illustrating the top three enriched GO-terms and the associated genes of the enhanced (**b**) and diminished (**c**) genes in U-CH11R. The fold change of gene expression is indicated by the color, the size of each node represents the number of linked genes to each term. **d** Rank-rank hypergeometric overlap (RRHO) heatmap for matched and unmatched dataset comparison used to identify significantly concordant transcriptional profiles from two independently conducted microarray gene expression analyses. RRHO was applied to the matched and unmatched data of primary and recurrent chordoma cell lines revealing high overlap between both datasets. The -log_10_ transformed hypergeometric p-value indicates the strength of enrichment (refer to legend). Concordantly up- and downregulated genes are located in A and D, respectively, disconcordantly expressed genes are in B and C. **e** Gene set enrichment analysis of the concordantly upregulated genes in recurrent chordoma cell lines. The top six enriched GO-terms and associated genes are given

Expression differences between U-CH11 and U-CH11R were identified by microarray gene expression analysis. More than 3000 genes were found to be significantly differently expressed (Fig. 3a) and expression differences of selected genes were validated by qRT-PCR (Additional file 1 (Additional Figure 3)).

*CALD1* (6968-fold), *COL1A2* (3054-fold), and *POSTN* (2995-fold) are the top overexpressed genes in U-CH11R versus U-CH11. Also, several oncogenes such as *BCL2* (11.5-fold), *BRAF* (9.8-fold), *KRAS* (4.9-fold), and *SRC* (2.8-fold) are significantly stronger expressed in the recurrent chordoma cell line (Additional file 3).

To address the functional potentials, GO annotations (MSigDB C5: GO) were applied to the overexpressed genes in U-CH11R. GSEA revealed that these genes are associated with extracellular matrix structural constituent, extracellular structure organization and cell matrix adhesion (Fig. 3b), bone morphogenesis, bone development, and skeletal system morphogenesis (Additional file 4).

The most down regulated genes in U-CH11R are *GABRA2* (− 1533-fold), *lnc-TPTE3* (− 1159-fold) and *GLYAT* (− 1075.2-fold) (Fig. 3a). Associated GO-terms included RNA splicing, RNA splicing via transesterification reactions and spliceosomal complex (Fig. 3c). A high proportion of diminished genes mapped to chromosome 1p and chromosome 8q, when assigned to the corresponding cytogenetic position (MSigDB C1; Additional file 4). Therefore, we assume that the decreased gene expression levels are partially related to the genomic aberrations, observed in the aCGH analyses of U-CH11 and U-CH11R (Fig. 1d). By contrast, the upregulation of gene expression in U-CH11R seems to occur independently of chromosomal alterations.

### U-CH11 and U-CH11R serve as a representative model of tumor recurrence in chordoma

To further confirm the identified expression differences between the matched primary and recurrent chordoma cell lines U-CH11 and U-CH11R, the analysis was extended to an additional microarray gene expression data set including unmatched primary (*n* = 3) and recurrent (n = 3) sacral chordoma cell lines.

In order to examine the statistically significant overlap in the gene expression signatures in matched and unmatched analyses the RRHO-approach was used [27]. A significant enrichment of gene overlap was detected in the on-diagonal extremes of the RRHO-plot clearly indicating a high amount of shared gene expression patterns (Fig. 3d). The concordantly upregulated genes had the greatest overall significance (hypergenomic *p*-value (HP) 10^− 37^). GSEA of these genes revealed an association to the GO-terms extracellular structure organization, cell junction organization and cell junction assembly (Fig. 3e). Furthermore, a linkage to extracellular matrix organization and degradation of the extracellular matrix pathways was observed by applying the MSigDB Reactome gene set C2 (Additional file 5). Interestingly, a high proportion of *HOX* genes was included within the enhanced expressed genes in recurrent chordoma cell lines, e.g., *HOXA-AS3*, *HOXB7,* and *HOXB13*. An increased overlap was also detected in concordantly downregulated genes. These results support the hypothesis that primary and recurrent chordoma cell lines have distinct gene expression signatures, reflected by the U-CH11 and U-CH11R model.

### The miR196a-5p is involved in the regulation of developmental *HOX* genes

**Fig. 4:**
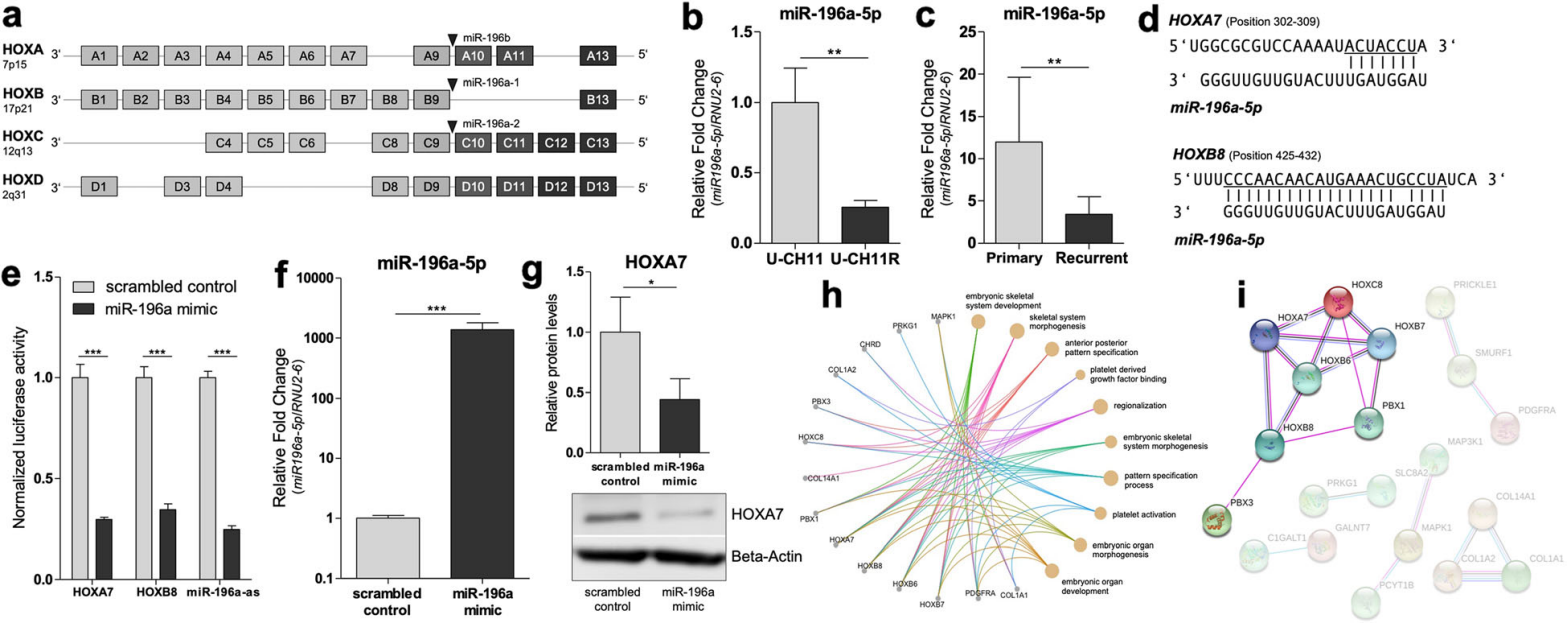
The HOX/PBX network is dysregulated by diminished miR-196a-5p levels in recurrent chordoma cell lines. **a** Scheme of the *HOX* clusters and miRNAs of the miR-196 family located within the cluster. Micro-RNA-196a-5p expression levels of matched (**b**) and unmatched (**c**) primary and recurrent chordoma cell lines. Relative fold change levels are given on the y-axis. (**d**) miR-196a-5p target sites (underlined nucleotides) in the 3’UTRs of *HOXA7* and *HOXB7* predicted by TargetScan 7.2. (**e**) Response to miR-196a-5p in U-CH1 cells. Firefly luciferase reporters containing either the complementary site of *HOXA7* and *HOXB8* or the perfect antisense sequence of miR-196a were co-transfected with a miR-196a mimic or a scrambled control, respectively. Firefly luciferase activity was normalized to Renilla luciferase activity. (**f**) Overexpression of miR-196a-5p in U-CH1 cells confirmed by qRT-PCR. (**g**) The effect of miR-196a-5p on HOXA7 levels in U-CH1 cells assessed by Western blot analysis. Experiments were performed in biological triplicates. T-tests were performed to determine statistical significance (*p < 0.05, **p < 0.01, ***p < 0.001). (**h**) GO-term analysis of the predicted target genes (*n* = 50) illustrated as cnetplot. (**i**) STRING analysis of the predicted miR-196a-5p target genes (n = 50) revealed an interaction network between HOX transcription factors and PBX co-factors

Due to the high proportion of differentially expressed *HOX* genes, the analysis was extended to scientifically proven regulators of the *HOX* clusters. Several genes encoding miRNAs, including the miR-196a-5p and miR-196b-5p, are known to be located within the clusters and to regulate *HOX* mRNA levels during embryonic development (Fig. 4a) [32, 33]. Significantly reduced miR196a-5p levels (− 4-fold) were detected in U-CH11R versus U-CH11 (Fig. 4b), correlating inversely with higher *HOX* expression levels. This finding was confirmed by comparing the miR196a-5p levels of unmatched primary (*n* = 3) and recurrent (n = 3) sacral chordoma cell lines (Fig. 4c). The predicted binding sequence of miR-196a-5p within the 3’UTR of the two selected *HOX* genes *HOXA7* and *HOXB8* mRNA is depicted in Fig. 4d.

To confirm these *HOX* genes as regulatory targets of miR-196a-5p, dual luciferase reporter assays were performed in U-CH1 chordoma cells (Fig. 4e) and HEK239T (Additional file 1 (Additional Figure 4)). Luciferase reporter plasmids that carry human HOXA7–3’UTR, HOXB8–3’UTR or the antisense sequence of miR-196a-5p were co-transfected with either miR-196a-5p mimics or scrambled controls. In U-CH1 overexpression of miR-196a-5p significantly reduced HOXA7–3’UTR and HOXB8–3’UTR reporter activity to 29.7 and 34.5%, respectively, compared to scrambled control. Similar results were observed for the mir-196a-antisense reporter (24.7%) which served as a positive control.

Furthermore, the effect of miRNA-196a-5p on HOXA7 protein levels was determined performing miRNA mimic transfections in the cell line U-CH1. Quantitative-RT-PCR experiments of the transfected cells revealed miRNA-196a-5p expression levels of > 1000-fold compared to control (Fig. 4f).

As expected, overexpression of miR-196a-5p significantly reduced HOXA7 levels to 44.3% (Fig. 4b) Taken together, these results suggest that *HOX* genes are downstream regulatory targets of the miR-196a-5p in chordoma.

In silico analysis was performed to evaluate further targets of the miR-196a-5p. Therefore, all identified upregulated genes in recurrent chordoma cell lines were compared with the predicted miR-196a-5p target genes. An intersection of 50 potential target genes was found (Additional file 6). GO enrichment analysis of these genes suggested an implication in early developmental processes, including the GO-terms embryonic skeletal system, skeletal system morphogenesis and anterior posterior pattern specification (Fig. 4h). STRING database analysis constructed an interaction network between HOX and PBX transcription factors (Fig. 4i), suggesting vulnerability towards HOX-PBX network inhibition in recurrent chordomas.

### Inhibition of dimer formation between PBX and HOX factors causes cell death in chordoma via enhanced cFOS expression

**Fig. 5:**
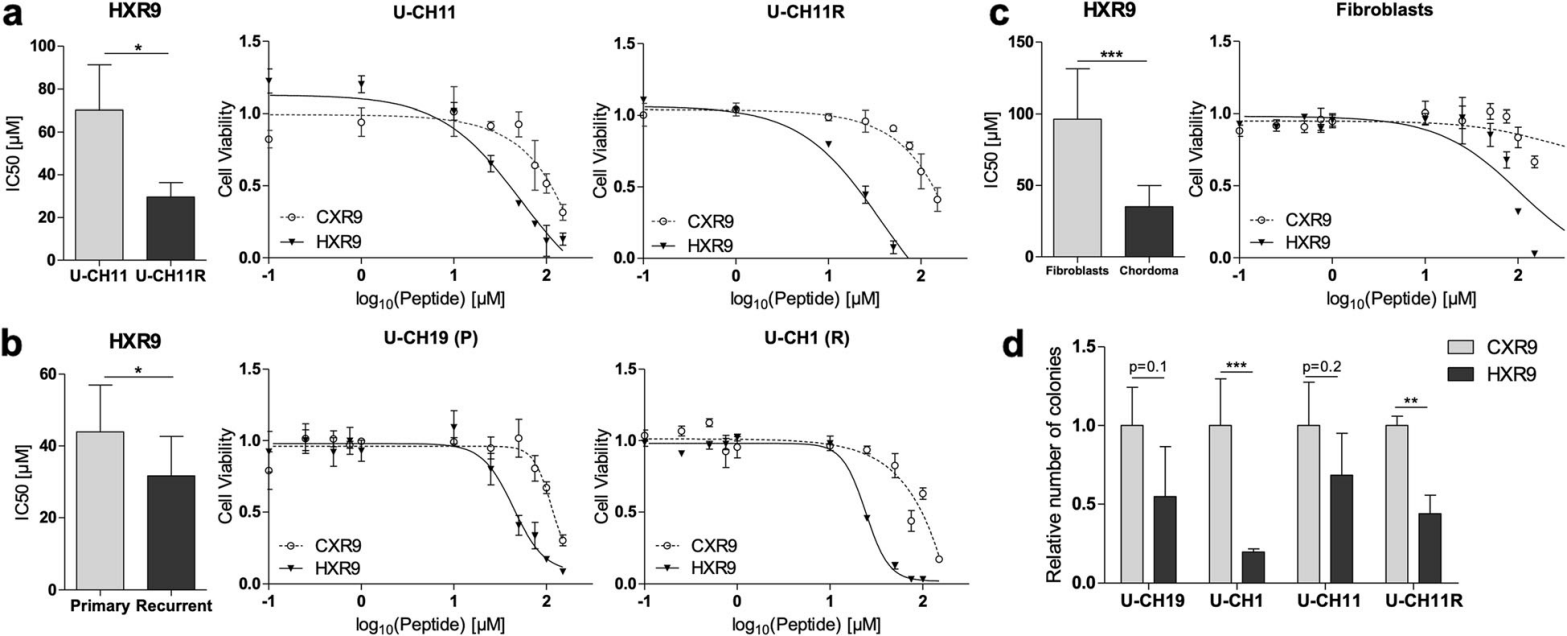
Recurrent chordoma cell lines are susceptible towards HOX/PBX inhibition. (**a**) Mean IC50 ± SD of HXR9 in U-CH11 (70.26 μM ±17.2) compared to U-CH11R (29.59 μM ±5.5) and (**b**) unmatched primary (n = 3, 43.91 μM ±12.3) versus recurrent (n = 3, 31.65 μM ±10.4) chordoma derived cell lines. Representative inhibition curves of one representative replicate of the cell lines treated with HXR9 (solid curves) and CXR9 (dotted curves) are depicted. (**c**) Overall response to HXR9 in chordoma (*n* = 8; 40.82 μM ±17.2) in comparison to fibroblasts (n = 3; 108.3 μM ±19.9). The inhibition curves of one representative example of fibroblasts are given. (**d**) Colony formation assay. Values are normalized to the mean amount of colonies after CXR9 treatment (n = 3). Statistical differences were determined by Student’s t-tests (*p < 0.05, **p < 0.01, ***p < 0.001)

HXR9 is a small peptide shown to interfere in dimer formation of PBX and HOX proteins by mimicking the PBX binding site of several HOX proteins [20, 34, 35]. Therefore, chordoma cells were treated with this peptide to elucidate the effect of HOX-PBX interference regarding cell survival in chordoma. HXR9 substantially reduced the viability of all cell lines tested. Significantly lower concentrations of HXR9 were required for U-CH11R (*n* = 3; mean IC50 = 29.59 μM ± 5.5) compared to U-CH11 (n = 3; mean IC50 = 70.26 μM ± 17.2; Fig. 5a). In unmatched recurrent chordoma cell lines, a mean IC50 value of 31.65 μM for HXR9 was computed which was significantly lower than the mean IC50 value of primary chordomas (43.91 μM; Fig. 5b). Significantly higher levels of the control peptide CXR9 were required to reduce the cells’ viability. No bottom plateau was reached and IC50 estimations revealed values of > 100 μM in all cell lines tested (Fig. 5a and b). Furthermore, compared to fibroblasts, chordoma cells were markedly more susceptible towards HXR9 treatment, substantiating the therapeutic potential of HOX-PBX-network inhibition (Fig. 5c). Colony formation assays of the primary chordoma cell lines U-CH19 and U-CH11 as well as the recurrent lines U-CH1 and U-CH11R were performed to test if a treatment with HXR9 has an impact on the cell lines’ ability to form colonies in soft agar. In the recurrent cell lines U-CH1 and U-CH11R the amount of colonies was significantly reduced to 20% (*p* < 0.01) and 44% (p < 0.01), respectively. In the primary chordoma cell lines (U-CH19 and U-CH11) this reduction could only be observed in trend (Fig. 5d).

**Fig. 6:**
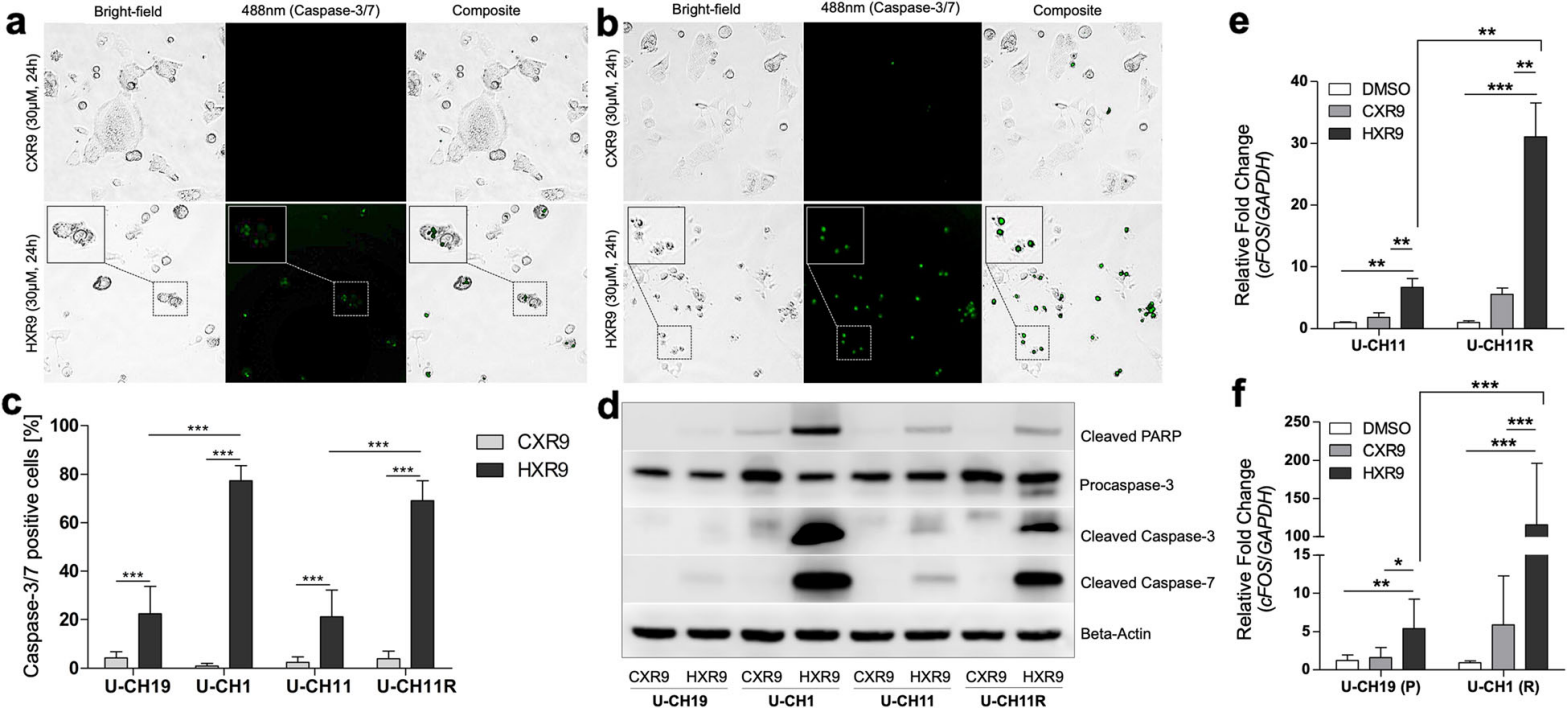
HXR9-induced apoptosis is mediated via cFOS. Activation of cleaved Caspase-3/7 (488 nm; green channel) in U-CH11 (**a**) and U-CH11R (**b**) treated for 24 h with 30 μM CXR9 or HXR9. The percentage of cleaved Caspase 3/7 positive cells following each treatment was determined by cell counts of representative image sections (*n* = 12; **c**). Immunoblots showing cleaved PARP, Procaspase 3, cleaved Caspase-3 and cleaved Caspase-7 in chordoma cell lines treated with 30 μM of HXR9 or CXR9 for 24 h (**d**). *CFOS* expression levels in response to treatment with HXR9 or two control compounds (CXR9 and DMSO) in U-CH1 versus U-CH19 (**e**) and U-CH11 compared to U-CH11R (**f**) cell lines quantified by qRT-PCR and normalized against *GAPDH*. Gene expression experiments were performed in technical and biological triplicates. Statistical differences were determined by Student’s t-tests (*p < 0.05, **p < 0.01, ***p < 0.001)

To investigate whether chordoma cells are undergoing apoptosis due to HXR9 treatment, activation of Caspase 3/7 was measured using a fluorescent Caspase-3/7 in vitro detection assay and compared to CXR9 treated cells. Figure 6 shows bright-field, corresponding fluorescent cleaved Caspase-3/7 staining and composite pictures of U-CH11 (a) and U-CH11R (b) cells treated with HXR9 or CXR9 following a 24 h treatment with 30 μM CXR9 or HXR9 (Staining of U-CH19 and U-CH1: Additional file 1 (Additional Figure 5)).

In response to HXR9 treatment significantly more cells appeared Caspase-3/7 positive in the recurrent line U-CH1 (77.5%) compared to the primary chordoma cell line U-CH19 (22.5%) and in U-CH11R (69%) compared to the corresponding line U-CH11 (21%), fitting their lower sensitivity to the peptide (Fig. 6c). In comparison, positive cell rates were < 5% due to CXR9 treatment in all cell lines tested. Similar results were obtained by immunohistochemical staining of cleaved Caspase-3 of chordoma cell lines treated for 4 h with 30 μM of HXR9 and CXR9. (Additional file 1, Figure 6).

Induction of apoptosis was further validated by Western blot analysis of HXR9 and CXR9 (30 μM, 24 h) treated cell lines. Strong signals of cleaved PARP, cleaved Caspase-3 and cleaved Caspase-7 were detected in the recurrent chordoma cell lines U-CH1 and U-CH11R subsequent to HXR9 treatment confirming the previous results observed in the NucView Caspase-3/7 assay, whereas the signals were weak in the primary cell lines U-CH19 and U-CH11. Additionally, HXR9 induced cleavage of poly (ADP-ribose) polymerase (PARP) in all cell lines compared to CXR9 control further substantiating induction of apoptosis (Fig. 6d).

Recently, it has been suggested that the HOX/PBX inhibition-induced apoptosis is mediated via cFOS [20]. Therefore, *CFOS* expression analyses in response to a four-hour treatment with 30 μM of the peptides and a vehicle control (DMSO) were conducted and normalized against *GAPDH* and *ACTB* (Fig. 6e; Additional file 1 (Additional Fig. 7)). HXR9 significantly increased *CFOS* levels compared to the control compounds CXR9 and DMSO. In line with the higher susceptibilities towards HXR9 in recurrent chordoma cell lines, a significant increase (4.6-fold) of *CFOS* mRNA levels was observed in U-CH11R compared to U-CH11 (Fig. 6e). Additionally, *CFOS* expression enhancement was considerably stronger (115-fold) in U-CH1 compared to U-CH19, which served as representative examples of a primary and recurrent chordoma cell line (Fig. 6f). These results suggest that the HXR9 induced apoptosis is mediated via enhanced *CFOS* expression in chordoma.

## Discussion

Chordomas are rare tumors with a high tendency to recur. Due to their low incidence, however, in vitro cell culture models have been limited to the investigation of tumorigenesis and metastasis so far. Consequently, an understanding of the cellular and genetic features of relapsed chordomas and their associated vulnerabilities has been missing. The chordoma cell lines derived from the same patient at the time of diagnosis (U-CH11) and following loco-regional recurrence (U-CH11R) serve as a unique cell line progression model to overcome this deficiency. Notably, the cell lines were established from patient samples that were not exposed to chemotherapeutic drugs and radiation. Therefore, an influence on the tumor biology in response to a systemic tumor treatment is excluded.

Both cell lines exhibited a very similar morphology and immunocytochemical profile that largely coincided with the findings in the corresponding tissues. However, the recurrent chordoma and derived cell line U-CH11R had a higher Ki-67 index and a shorter PDT in cell culture than the primary tumor and its corresponding cell line U-CH11. These findings implicate substantial differences between the primary and the recurrent tumor detectable in the tissues and being reflected in the corresponding cell lines.

The aCGH profile of both cell lines was almost congruently altered, including losses of chromosomes 3p, 22q and on chromosome 10, which are frequently reported in chordomas [36]. The profound similarities between U-CH11 and U-CH11R show that the cells of both lines are clonally related. Thus, it seems most likely that cells of the primary and recurrent chordoma emanated from a common progenitor but additionally accumulated a few individual aberrations over time, e.g. loss of 1p in U-CH11R. Loss of 1p is associated with tumor progression and recurrence in neuroblastoma, with distant recurrence and poor prognosis in a range of solid malignancies [37]. Recently, the 1p36 locus received growing attention in various cancer entities, suggesting tumor suppressor genes to reside within this area [38]. Riva et al. suggested the genes *CASP9*, *EPH2A,* and *DVL1*, located on 1p36.32–36.11, to contribute to oncogenesis in chordoma [39]. In a series of skull base chordomas, loss of heterozygosity (LOH) at 1p36 was observed in 75% of the cases and was correlated with poor prognosis [40]. Therefore, loss of 1p could be a conductive feature to drive chordoma cells towards a more aggressive phenotype.

We detected a high amount of exonic mutations in the primary tumor tissue being conserved in U-CH11 and U-CH11R. Numerous variants were only detected within the primary chordoma tissue and U-CH11R, but not in U-CH11. Hence, it can be assumed that cells that gave rise to the recurrent chordoma and the derived cell line U-CH11R were already present in the primary tumor. Interestingly, U-CH11R harbors all mutations that were predicted deleterious implicating a contribution to tumor progression. Among them *ERCC2*, encoding the Xeroderma Pigmentosum Protein, is frequently altered in different types of cancer (reviewed in [41]). *ABBC1* refers to the multidrug resistance-associated protein 1. Genetic variations in *ABCC1* might affect drug disposition or efficacy [42].

To explore how the genomic features contribute to differential gene expression in U-CH11 and U-CH11R, gene expression patterns were compared. GSEA of the downregulated genes in U-CH11R revealed an association to RNA-Splicing. Although pathologically altered splicing events and enhanced dependency on the spliceosome in chordoma are yet barely investigated, gene expression alterations affecting components of the splicing machinery have been recently detected in a widespread of other cancer types and are suggested to promote cancer development and treatment resistance. Promising strategies by which pathologic splicing events may be modulated for cancer therapy are currently under investigation and may be transferred to chordoma in the future [43].

Moreover, a high proportion of genes that appeared to be downregulated in U-CH11R mapped to 1p, such as the above-mentioned genes *CASP9* and *DVL1*. The upregulated genes in U-CH11R, however, could not be explicitly linked to the chromosomal alterations, suggesting further key regulatory mechanisms. The highest upregulation was observed for genes referring to the structural proteins caldesmon (*CALD*) and collagen type I alpha 2 (*COL1A2*). Caldesmon and COL1A2 are found at high levels in various gastrointestinal tumors carcinoma [44–47]. To prove whether the expression profile of U-CH11R is conferrable to other recurrent chordoma cell lines, a second set of gene expression data of unmatched primary and recurrent chordoma cell lines was included. Recently, it was shown that clival and sacral chordoma cell lines differ substantially in their expression profiles [13]. Thus, we only included cell lines derived from sacral chordomas. The RRHO method was applied to identify patterns of concordant transcriptional changes in the matched (U-CH11 vs. U-CH11R) and the unmatched dataset (primary vs. recurrent chordoma cell lines). We found the highest enrichment of gene overlap close to the on-diagonal extremes indicating that strongly differentially expressed genes are not explicable by intrapersonal effects but can be regarded as general expression differences between primary and recurrent chordoma cell lines [27]. Interestingly, several *HOX* genes appeared to be highly upregulated in recurrent chordoma cell lines. Shah et al. recently suggested that aberrant *HOX* gene expression might contribute to oncogenesis by promoting the activation of anti-apoptotic pathways and being involved in cell invasion, EMT, DNA damage repair and proliferation [48]. Moreover, a transcriptome analysis revealed numerous *HOX* genes to be overexpressed in recurrent glioblastomas [49]. In chordomas, genes of the *HOXA* cluster have been reported to be super-enhancer associated in vitro and in vivo [3]. Furthermore, *HOXC8* overexpression has been shown to promote proliferation, colony formation, and cell invasion in the recurrent chordoma derived cell lines U-CH1 and U-CH2 [50]. We therefore assume a role of HOX factors in chordoma relapse. The genes *HOXB7*, *HOXB13,* and *HOXA-AS3* were detected among the upregulated genes in recurrent chordomas. Their overexpression has frequently been observed in a broad range of solid tumors, such as gastric-, prostate-, and small cell lung cancer and is associated with poor prognosis [51–53]. Due to the large number of dysregulated *HOX* genes in recurrent chordoma cell lines, we assume the same underlying key regulator. We therefore assessed the expression of the miRNA gene family *MIR196*, located within the *HOX* cluster [33]. The miR-196a and miR-196b are known to regulate *HOX* mRNA levels during embryonic development and to function as either oncogenes or tumor suppressors in a variety of tumor entities. A tumor suppressive role was suggested in osteosarcoma, melanoma, and breast cancer [54–56]. Compatible with the enhanced *HOX* mRNA levels, we observed significantly lower miR-196a levels in recurrent chordoma cell lines. By STRING analysis, we identified an interaction network between HOX and PBX factors in recurrent chordomas, potentially regulated by the miR-196a. The mechanism of HOX-PBX dimerization and its inhibition using the small peptide HXR9 have previously been described in several tumor entities [35]. Therefore, we inhibited the HOX-PBX interaction using HXR9. This induced cell death in all chordoma cell lines analyzed and reduced the ability of the cells to form colonies. Recurrent chordoma derived cell lines were significantly more susceptible towards HOX/PBX-inhibition than primary cell lines. In line with this, a higher increase of *CFOS* expression following HXR9 treatment was detected in recurrent chordoma cell lines [57].

## Conclusion

Taken together, our results suggest that transcriptomic reprogramming occurs during chordoma recurrence, which does not derive from genomic events. The upregulation of the HOX-PBX network is, at least in parts, regulated by the miR-196a in recurrent chordoma and can be disrupted using the peptide HXR9 in order to induce apoptosis.

## Supplementary Information

**Additional file 1: Figure 1**. Results of mycoplasma PCR of cell lines included in the analyses. (−) negative control; (+) two different mycoplasma positive controls showing the expected amplicon (~ 500 bp). All cell lines tested were negative. **Figure 2**. Immunohistochemistry (IHC) of the primary and the relapsed sacral chordoma in comparison to the derived cell lines. IHC results were interpreted as follows: “no immunoreactivity detected” (−), “immunoreactivity ≤ 30%” (+), “immunoreactivity > 30% and < 70%” (++), “immunoreactivity in ≥ 70%” (+++) of the total number of chordoma cells analyzed. HE: hematoxylin/eosin; EMA: epithelial membrane antigen. NOS: not otherwise specified. **Figure 3**. Validation of gene expression differences between U-CH11 and U-CH11R. Expression levels of selected genes were quantified by qRT-PCR analyses and normalized against *GAPDH*. Experiments were performed in technical triplicates and at least in biological duplicates and Student’s t-tests were performed to determine statistical significance (*p < 0.05, **p < 0.01, ***p < 0.001). **Figure 4**. Dual Luciferase reporter assay of miR-196a-5p in HEK293T cells. Firefly luciferase reporters containing either the complementary site of *HOXA7* and *HOXB8* or the perfect antisense sequence of miR-196a were co-transfected with a miR-196a mimic or a scrambled control, respectively. Firefly luciferase activity was normalized to Renilla luciferase activity. Experiments were performed in technical and biological triplicates and Student’s t-tests were performed to determine statistical significance (*p < 0.05, **p < 0.01, ***p < 0.001). **Figure 5**. Activation of Caspase-3/7 in U-CH19 and U-CH1. Activation of cleaved Caspase-3/7 (green channel) in U-CH19 (a) and U-CH1 (b) treated for 24 h with 30 μM CXR9 or HXR9 investigated by EarlyTox Caspase-3/7 NucView 488 assays. **Figure 6**. Induction of apoptosis in HXR9 treated chordoma cell lines assessed by immunocytochemistry of cleaved caspase-3 (red staining). Cell lines were treated for 4 h with 30 μM HXR9 or the control peptide CXR9. No cleaved caspase-3 positivity was observed in response to CXR9 treatment. **Figure 7**. Expression levels of *cFOS* normalized against *ACTB*. *CFOS* expression levels in response to treatment with HXR9 or two control compounds (CXR9 and DMSO) in U-CH11 versus U-CH11R (a) and U-CH19 compared to U-CH1 (b) cell lines quantified by qRT-PCR and normalized against *ACTB*. Gene expression experiments were performed in technical and biological triplicates. Statistical differences were determined by Student’s t-tests (*p < 0.05, **p < 0.01, ***p < 0.001). **Table 1**. Antibodies used for immunostainings of chordoma cell lines and tissue. **Table 2**. STR profiles of U-CH11R and U-CH11. **Table 8**. Base-level expression of selected genes based on microarray gene expression data. Mean raw intensity values and standard deviations are given.

**Additional file 2: Table 3**. Filtered exonic variants in the primary tumor tissue (11PT) and the cell lines U-CH11 and U-CH11R.

**Additional file 3: Table 4**. List of up- and downregulated genes in U-CH11R versus U-CH11 based on the microarray gene expression analysis.

**Additional file 4: Table 5**. GSEA of differentially expressed genes in U-CH11R and U-CH11

**Additional file 5: Table 6**. GSEA of RRHO Plot Quadrant A.

**Additional file 6: Table 7**. List of potential miR-196a-5p target genes in recurrent chordoma cell lines.

## Data Availability

The dataset(s) supporting the conclusions of this article is (are) included within the article (and its additional file(s)). Used software is given in the methods section.

## Abbreviations

ABCC1: ATP Binding Cassette Subfamily C Member 1
ABCF3: ATP Binding Cassette Subfamily F Member 3
aCGH: Array comparative genomic hybridization
AF: Allele frequency
ANNOVAR: Annotate variation
Asp: Asparagine
CALD1: Caldesmon 1
cDNA: Complementary deoxyribonucleic acid
COL1A2: collagen type I alpha 2 chain
Ct: Cycle threshold
DMSO: Dimethyl sulfoxide
EHD3: EH Domain Containing 3
EMA: epithelial membrane antigen
FC: Fold change
Gly: Glycin
GO: Gene ontology
GSEA: Gene set enrichment analysis
HFF: Human foreskin fibroblasts
HOX: Homeobox
HP: Hypergenomic *p*-value
LOH: loss of heterozygosity
MCCC1: Methylcrotonoyl-CoA Carboxylase 1
miRNA: Micro ribonucleic acid
MSigDB: Molecular signature database
MTS: 3-(4,5-dimethylthiazol-2-yl)-5-(3-carboxymethoxyphenyl)-2-(4-sulfophenyl)-2H-tetrazolium)
PBX: Pre-B cell leukemia transcription factor
PCR: Polymerase chain reaction
PDT: Population doubling time
POSTN: Periostin
qRT-PCR: Quantitative real time polymerase chain reaction
RNA: Ribonucleic acid
RRHO: Rank-rank hypergeometric overlap
SNP: Single nucleotide polymorphism
SNV: Single nucleotide variant
STR: Short tandem repeat
UTR: Untranslated region
