# Supplementary material for: Molecular features and vulnerabilities of recurrent chordomas
Source: J Exp Clin Cancer Res. 2021 Jul 30;40:244. doi: 10.1186/s13046-021-02037-y (PMC8325178; doi:10.1186/s13046-021-02037-y)
Supplement: Supplementary file 1 — Additional file 1: Figure 1. Results of mycoplasma PCR of cell lines included in the analyses. (−) negative control; (+) two different mycoplasma positive controls showing the expected amplicon (~ 500 bp). All cell lines tested were negative. Figure 2. Immunohistochemistry (IHC) of the primary and the relapsed sacral chordoma in comparison to the derived cell lines. IHC results were interpreted as follows: “no immunoreactivity detected” (−), “immunoreactivity ≤ 30%” (+), “immunoreactivity > 30% and < 70%” (++), “immunoreactivity in ≥ 70%” (+++) of the total number of chordoma cells analyzed. HE: hematoxylin/eosin; EMA: epithelial membrane antigen. NOS: not otherwise specified. Figure 3. Validation of gene expression differences between U-CH11 and U-CH11R. Expression levels of selected genes were quantified by qRT-PCR analyses and normalized against GAPDH. Experiments were performed in technical triplicates and at least in biological duplicates and Student’s t-tests were performed to determine statistical significance (*p < 0.05, **p < 0.01, ***p < 0.001). Figure 4. Dual Luciferase reporter assay of miR-196a-5p in HEK293T cells. Firefly luciferase reporters containing either the complementary site of HOXA7 and HOXB8 or the perfect antisense sequence of miR-196a were co-transfected with a miR-196a mimic or a scrambled control, respectively. Firefly luciferase activity was normalized to Renilla luciferase activity. Experiments were performed in technical and biological triplicates and Student’s t-tests were performed to determine statistical significance (*p < 0.05, **p < 0.01, ***p < 0.001). Figure 5. Activation of Caspase-3/7 in U-CH19 and U-CH1. Activation of cleaved Caspase-3/7 (green channel) in U-CH19 (a) and U-CH1 (b) treated for 24 h with 30 μM CXR9 or HXR9 investigated by EarlyTox Caspase-3/7 NucView 488 assays. Figure 6. Induction of apoptosis in HXR9 treated chordoma cell lines assessed by immunocytochemistry of cleaved caspase-3 (red staining). Cell lines w [file 13046_2021_2037_MOESM1_ESM.pdf]

Molecular features and vulnerabilities of recurrent chordomas

Carolin Seeling[1], André Lechel[2], Michael Svinarenko[2], Peter Möller[1 *], Thomas F.E. Barth[1], and Kevin Mellert[1]

1. Institute of Pathology, University Hospital of Ulm, Ulm, Germany
2. Department of Internal Medicine I, University Hospital Ulm, Ulm, Germany

***Corresponding Author:**

Peter Möller, MD

Institute of Pathology, M23

University Hospital of Ulm

Albert-Einstein-Allee 11

D-89081 Ulm

Germany

Phone: +49-(0)731-50056320

Fax: +49-(0)731-50056384

email: peter.moeller@uniklinik-ulm.de

Additional Information

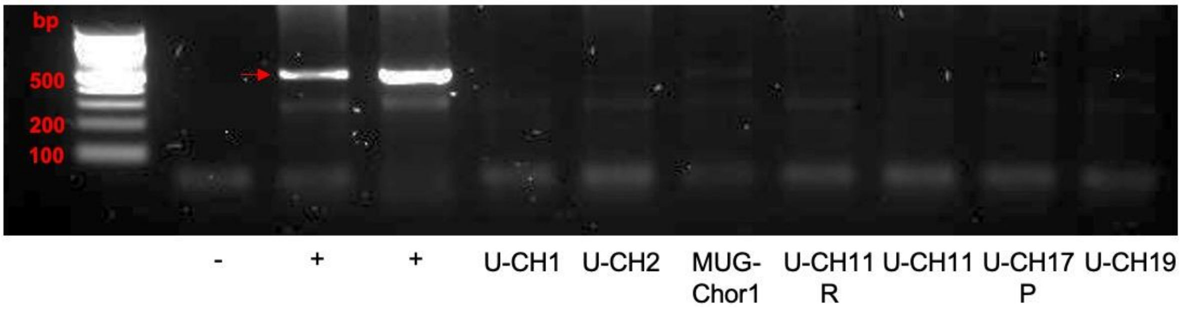

Additional figure 1: Results of mycoplasma PCR of cell lines included in the analyses. (-) negative control; (+) two different mycoplasma positive controls showing the expected amplicon (~500bp). All cell lines tested were negative.

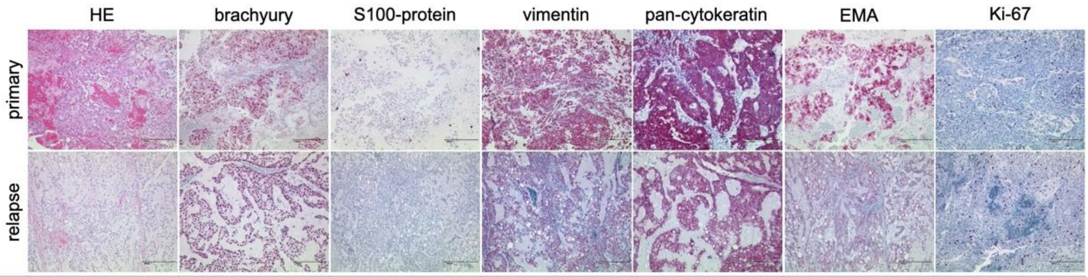

	HE	brachyury	S100-protein	vimentin	pan-cytokeratin	EMA	Ki-67
Primary tissue	NOS	+++ (nuclear)	-/+	+++	+++	++	1%
U-CH11		+++ (nuclear)	-	++	+++	+	<1%
Relapse tissue	NOS	+++ (nuclear)	-	++	+++	++	5%
U-CH11R		+++ (nuclear)	-	++	+++	+	5%

Additional figure 2: Immunohistochemistry (IHC) of the primary and the relapsed sacral chordoma in comparison to the derived cell lines. IHC results were interpreted as follows: “no immunoreactivity detected” (-), “immunoreactivity ≤ 30%” (+), “immunoreactivity > 30% and < 70%” (++), “immunoreactivity in ≥ 70%” (+++) of the total number of chordoma cells analyzed. HE: hematoxylin/eosin; EMA: epithelial membrane antigen. NOS: not otherwise specified.

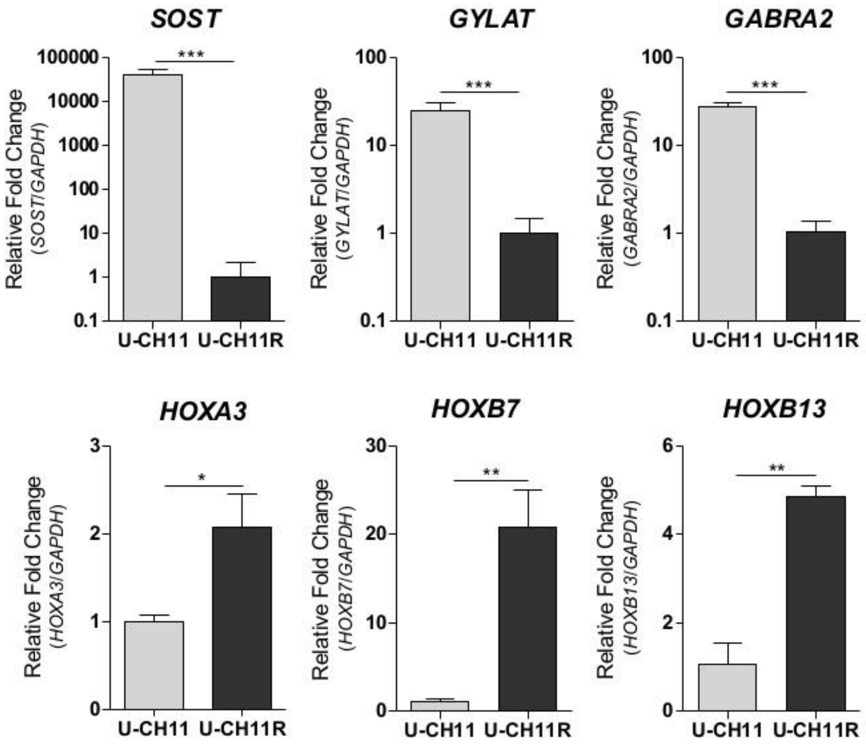

Additional figure 3: Validation of gene expression differences between U-CH11 and U-CH11R. Expression levels of selected genes were quantified by qRT-PCR analyses and normalized against *GAPDH*. Experiments were performed in technical triplicates and at least in biological duplicates and Student's t-tests were performed to determine statistical significance (*p<0.05, **p<0.01, ***p<0.001).

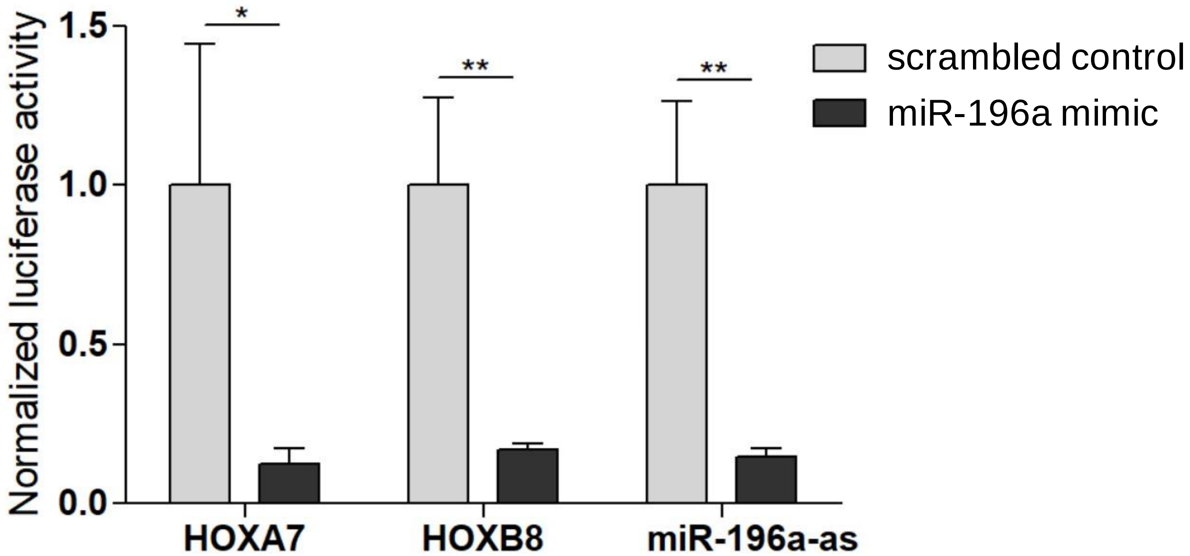

Additional figure 4: Dual Luciferase reporter assay of miR-196a-5p in HEK293T cells. Firefly luciferase reporters containing either the complementary site of *HOXA7* and *HOXB8* or the perfect antisense sequence of miR-196a were co-transfected with a miR-196a mimic or a scrambled control, respectively. Firefly luciferase activity was normalized to Renilla luciferase activity. Experiments were performed in technical

and biological triplicates and Student's t-tests were performed to determine statistical significance (*p<0.05, **p<0.01, ***p<0.001).

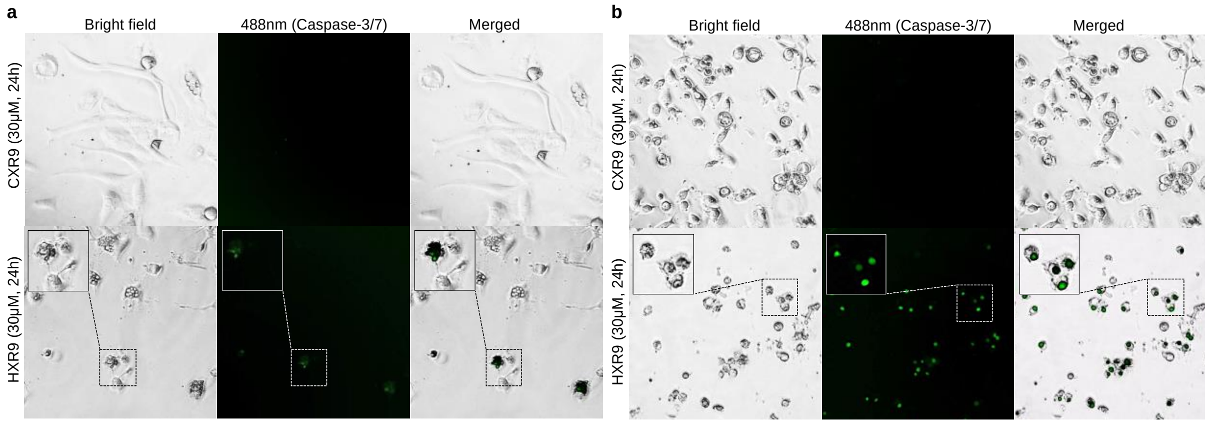

Additional figure 5: Activation of Caspase-3/7 in U-CH19 and U-CH1. Activation of cleaved Caspase-3/7 (green channel) in U-CH19 (**a**) and U-CH1 (**b**) treated for 24h with 30µM CXR9 or HXR9 investigated by EarlyTox Caspase-3/7 NucView 488 assays.

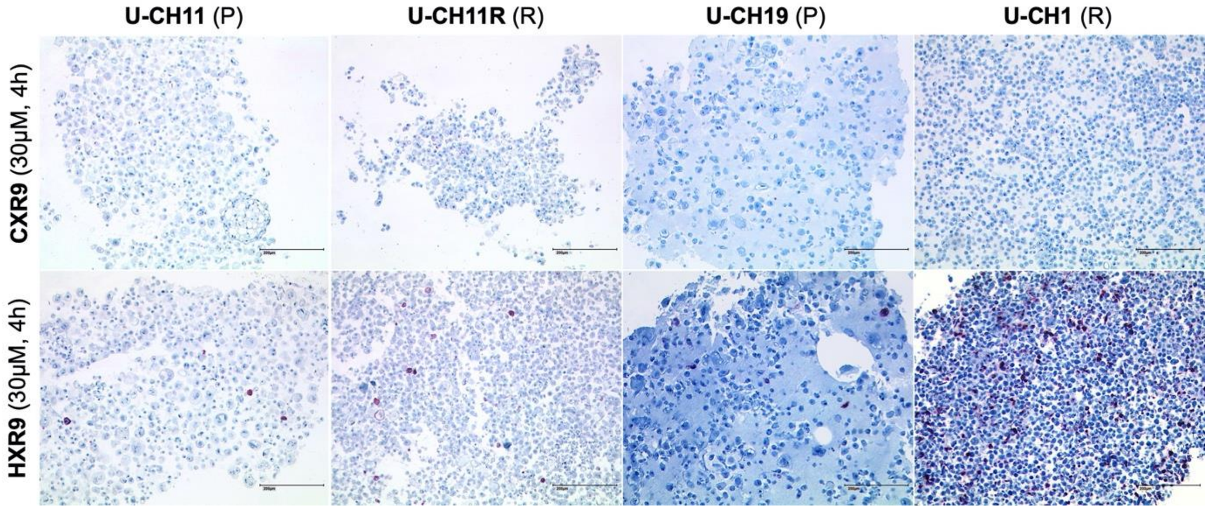

Additional figure 6: Induction of apoptosis in HXR9 treated chordoma cell lines assessed by immunocytochemistry of cleaved Caspase-3 (red staining). Cells were treated for 4h with 30µM HXR9 or the control peptide CXR9. Compared to HXR9 treatment, no cleaved Caspase-3 positivity was observed in response to CXR9.

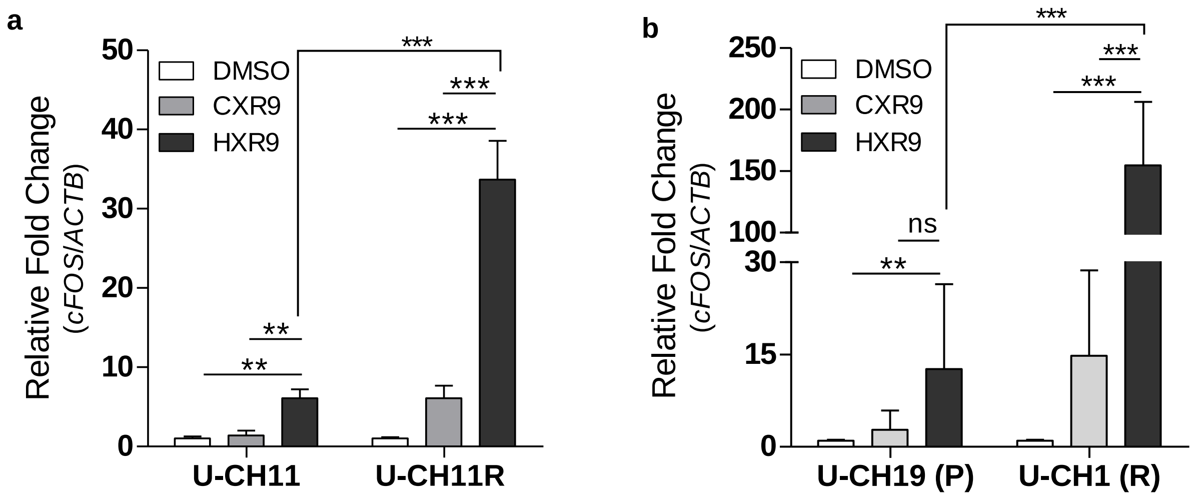

Additional figure 7: Expression levels of *cFOS* normalized against *ACTB*. *CFOS* expression levels in response to treatment with HXR9 or two control compounds (CXR9 and DMSO) in U-CH11 versus U-CH11R (**a**) and U-CH19 compared to U-CH1 (**b**) cell lines quantified by qRT-PCR and normalized against *ACTB*. Gene expression experiments were performed in technical and biological triplicates. Statistical differences were determined by Student's t-tests (*p<0.05, **p<0.01, ***p<0.001).

Additional table 1: Antibodies used for immunostainings of chordoma cell lines and tissue

Antibody	Clone	Company	Dilution
Brachyury	EPR18113	Abcam	1:4000
Cytokeratin	AE1 + AE3	Dako	1:100
Epithelial Membrane Antigen (EMA)	E29	Dako	1:500
Ki-67	MIB-1	Dako	1:200
S100-protein	Z0311	Dako	1:1000
Vimentin	VIM3B4	Dako	1:300
cleaved-caspase 3	polyclonal	Dako	1:500

Additional table 2: STR profiles of U-CH11 and U-CH11R

	AMEL		D13S317		D7S820		D16S539		Penta E		THO1	
**U-CH11**	X		14		9	10	9	11	7	14	6	9.3
**U-CH11R**	X		8	14	(9)	10	9	11	7	14	6	9.3

	D18S51		D3S1358		D8S11779		TPOX		CSF1PO		Penta D	
**U-CH11**	14		15		13	16	8		10	11	9	12
**U-CH11R**	14		15	16	13	16	8		10	11	9	12

Additional table 8: Base-level expression of selected genes based on microarray gene expression data. Mean raw intensity values and standard deviations are given.

GeneSymbol	U-CH11		U-CH11R		U-CH19		U-CH1	
	mean raw intensity	sd	mean raw intensity	sd	mean raw intensity	sd	mean raw intensity	sd
**References**								
MS4A1 (CD20; not expressed)	5.00	0.42	3.08	1.84	5.65	0.78	8.52	2.22
T (Brachyury; diagnostic chordoma marker)	2943.89	107.91	1071.99	330.40	4701.53	394.69	2218.19	436.73
GAPDH (housekeeping gene)	45973.58	16236.78	44591.84	18072.73	154013.26	12658.19	52720.23	8436.82
ACTB (ß-Actin; housekeeping gene)	28015.21	7603.27	34013.52	18369.19	32282.21	2325.51	21732.87	4452.30
FOS	11234.00	5180.33	965.12	201.18	1280.55	547.23	1698.53	540.58
**Different in U-CH11 vs U-CH11R**								
SOST	1897.31	535.03	3.24	1.05				
GLYAT	3213.43	436.36	2.81	1.42				
GABRA2	7896.56	5438.50	4.90	4.14				
HOXA3	30301.23	29563.06	136296.54	12150.52				
HOXB7	30.45	5.78	477.83	189.74				
HOXB13	30.22	3.21	308.18	25.71				
